# Increased household financial strain, the Great Recession and child health—findings from the UK Millennium Cohort Study

**DOI:** 10.1136/bmjopen-2016-015559

**Published:** 2017-03-09

**Authors:** Caoimhe McKenna, Catherine Law, Anna Pearce

**Affiliations:** UCL GOSH Institute of Child Health, London, UK

**Keywords:** SOCIAL MEDICINE, EPIDEMIOLOGY

## Abstract

**Background:**

There is a growing body of evidence associating financial strain (FS) with poor health but most of this research has been cross-sectional and adult-focused. During the ‘Great Recession’ many UK households experienced increased FS. The primary aim of this study was to determine the impact of increased FS on child health.

**Methods:**

We analysed the Millennium Cohort Study, a longitudinal study of children born in the UK between 2000 and 2002. Surveys at 7 years (T1, 2008) and 11 years (T2, 2012) spanned the ‘Great Recession’. Three measures of increased FS were defined; ‘became income poor’ (self-reported household income dropped below the ‘poverty line’ between T1 and T2); ‘developed difficulty managing’ (parental report of being ‘financially comfortable’ at T1 and finding it ‘difficult to manage’ at T2); ‘felt worse off’ (parental report of feeling financially ‘worse off’ at T2 compared with T1). Poisson regression was used to estimate risk ratios (RR), adjusted risk ratios (aRR) and 95% CIs for six child health outcomes: measured overweight/obesity, problematic behaviour as scored by parents and teachers, and parental reports of fair/poor general health, long-standing illness and bedwetting at T2 (N=13 112). In subanalyses we limited our sample to those who were above the poverty line at T2.

**Results:**

Compared with those who were not financially strained at both time points, children in households which experienced increased FS were at an increased risk of all unhealthy outcomes examined. In most cases, these increased risks persisted after adjustment for confounding and when limiting the sample to those above the poverty line.

**Conclusions:**

FS is associated with a range of new or continued poor child health outcomes. During times of widespread economic hardship, such as the ‘Great Recession’, measures should be taken to buffer children and their families from the impact of FS, and these should not be limited to those who are income poor.

**Strengths and limitations of this study**

- This research represents the first study using longitudinal data to look at a range of child health outcomes coinciding with the ‘Great Recession’.
- The UK Millennium Cohort Study is a large, nationally representative data set.
- Attrition is a common problem in cohort studies. We used survey weights to account for attrition between sweeps, but it remains possible the weights did not fully account for any bias.
- Our measures of financial strain were limited to the available data. All of the measures were self-reported and some were subjective. However, across all three measures there were associations with new or continued poor child health.

## Introduction

Financial strain (FS) occurs when resources are inadequate to meet needs and/or expectations. It incorporates inadequacy of resources, as well as subjective factors and other psychological influences.

There is a growing body of evidence associating FS with poor health but most is cross-sectional and adult focused.^1^ We identified only three previous studies looking specifically at household FS and child health outcomes. Hernandez and Pressler^2^ found that household FS was related to higher risk of overweight/obesity in adolescent girls. Jackson *et al*^3^ found that FS was associated with behavioural problems and lower ‘preschool ability’. Skafida *et al*^4^ found that mothers who transitioned from ‘living very comfortably’ to ‘finding it very difficult to cope’ on current income had children who consumed fewer fruit varieties and more unhealthy snacks, compared with those who remained financially comfortable.

The ‘Great Recession’ was a time when many households in the UK experienced increases in FS and families with children were disproportionately affected.^5^ The aim of this study was to determine if increases in household FS, over the period of the ‘Great Recession’, were associated with new or continued poor health among children. We also examined the relationship between increased FS and health in the non-income poor (ie, limited to families who were above the ‘poverty line’).

## Methods

Data examined were from the Millennium Cohort Study, a longitudinal study of children born in the UK between 2000 and 2002. Data were obtained from the UK Data Service, University of Essex, May 2012.^6–8^ To date, data are available at age 9 months, 3 years, 5 years, 7 years and 11 years. The information collected includes a wide range of parental reported sociodemographic and health factors. The original sample included 18 296 singleton children; 71.7% (N=13 112) of whom took in the most recent survey (11 years). At age 11 years 95% of main respondents were the child's natural mother. Surveys at age 7 years (T1, 2008) and 11 years (T2, 2012) spanned the ‘Great Recession’. Of those children who took part in the survey at T1, 1796 (13.1%) did not take part at T2.

### Exposure—increased FS

Three measures of increased FS, between T1 and T2, were defined:

‘Became income poor’*;* household income which was >60% of contemporary median at T1 (ie, above the poverty line) and ≤60% of contemporary median at T2 (ie, below the poverty line). Incomes were self-reported and Organisation for Economic Co-operation and Development (OECD) equivalised.^9^ The comparator group were those whose incomes were above the poverty line at T1 and T2.

‘Developed difficulty managing’*;* respondents were asked at T1 and T2 ‘how well would you say you are managing financially these days?’, (1) living comfortably, (2) doing alright, (3) just about getting by, (4) finding it quite difficult, (5) finding it very difficult. An increase in household FS was defined as going from a score of 1–3 at T1 to a score of 4 or 5 at T2. The comparator group were those who had a score of 1–3 at both time points.

‘Felt worse off’*;* respondents were asked at T2; ‘compared with the last interview would you say that you are better or worse off financially or about the same?’ An increase in FS was defined as being ‘a little’ or ‘a lot worse off’. The comparator group was those who felt their finances were ‘about the same’.

### Outcomes—poor child health at T2

Six dichotomous measures of poor health were examined at age 11 years (T2), thus analyses capture new or continued poor health between age 7 (T1) and 11 years (T2).

*Overweight/obesity;* children's height and weight were measured by a trained interviewer. Overweight, including obesity, was defined according to International Obesity Task Force cut-offs.^10^

*Problematic behaviour (borderline/abnormal Strengths and Difficulties Questionnaire (SDQ); teacher and parent scored);* the SDQ is a commonly used and standardised measure of child psychological well-being.^11^ We examined SDQs as scored by teachers and parents. Child problematic behaviour was defined as a total SDQ score of >11. SDQ scores above this level predict future psychiatric burden.^12^ ^13^ Teacher SDQ scores were only available for children living in England and Wales.

*General health score;* main respondents were asked to rate their child's general health on the following scale; (1) excellent, (2) very good, (3) good, (4) fair, (5) poor. A suboptimal child health score was defined as a response of 4 or 5. This is a widely used measure but there is some evidence that parents tend to overestimate the perceived general health of their children, compared with self-report.^14^

*Long-standing illness;* main respondents were asked ‘does your child have any physical or mental health conditions lasting, or expected to last, 12 months or more?. Parental-reported long-standing illness has been found to accurately reflect children's own report.^14^ ^15^

*Bedwetting;* parents were asked ‘which of these best applies to your child?’; (1) never wets the bed at night, (2) occasionally wets the bed, (3) wets the bed once/twice a week, (4) wets the bed 3+ times a week and (5) wears night-time pads. Any score >1 was considered unhealthy.

### Statistical analysis

Poisson regression was used to estimate risk ratios (RR), adjusted risk ratios (aRR) and 95% CIs^16^ for six poor child health outcomes at T2, according to three measures of increased FS. We repeated the analyses, limiting the sample to households which were above the ‘poverty line’ at T2 (ie, household income >60% of contemporary median). All analyses were adjusted for becoming a lone parent (ie, two parent household at T1 and one parent household at T2), ethnicity (main respondent white British/Irish, other), maternal level of education at 9 months (degree level or above) and parental age (continuous variable, years). Children who took part at the age 11 survey (T2) were less likely to be living in poverty when the child was age 7 (T1) than those who did not take part (29.3% vs 43.4%, p=<0.01). Analyses were conducted in Stata/SE13 (Stata Corporation, Texas, USA), using ‘svy’ commands to account for clustered sampling and attrition at T2.

Differences between the baseline sociodemographic and health characteristics of households which experienced increased FS, and their comparator groups, were assessed using χ^2^ for comparison of proportions, student's t-test for normally distributed continuous variables or Mann-Whitney U test for non-normal continuous variables. There was no consistent evidence of effect modification by gender so analyses shown are for both genders combined.

## Results

Table 1 summarises and compares the demographic and health characteristics of children in households which experienced an increase in household FS, alongside their comparator groups, at T1. At baseline (7 years, T1), those households which reported increased FS tended to have lower levels of household employment and maternal degree level education, as well as higher levels of lone parenthood, more children in the household and a main respondent who was younger and less likely to be of white British/Irish ethnicity. Children in these households also tended to have worse health at baseline, with higher levels of problematic behaviour, suboptimal general health scores and higher rates of longstanding illness.

**Table 1:** Summarising and comparing the baseline (T1) sociodemographic and child health characteristics in households which experienced increased household financial strain (T1–T2) and their comparator groups

	Became income poor n=507	Stayed non-poor n=7895	p Value	Developed difficulty managing n=1127	Did not report difficulty managing n=9142	p Value	Felt ‘worse off” n=4681	Felt ‘the same’ n=4533	p Value
	N(%)/avg (95% CI)	N(%)/avg (95% CI)		N (%)/avg (95% CI)	N(%)/avg (95% CI)		N(%)/avg (95% CI)	N(%)/avg (95% CI)	
**Sociodemographics at T1 (7 years)**									
Mean age of main respondent (years)	31 (30.5 to 31.5)	37.6 (37.5 to 37.7)	<0.01†	35.6 (35.3 to 36.0)	36.5 (36.4 to 36.6)	<0.01†	36.9 (36.7 to 37.0)	36.4 (36.2 to 36.6)	<0.01†
Mother degree level education+(at 9 months)	4 (0.9%)	2093 (23.5%)	<0.01	125 (8.4%)	1934 (19.2%)	<0.01	760 (13.8%)	768 (15.3%)	0.4
Anyone in the household employed	430 (86%)	7112 (88.8%)	<0.01	940 (83.2%)	8096 (88.3%)	<0.01	3634 (85.2%)	3683 (88.6%)	<0.01
Lone parent household	100 (17.4%)	788 (10.9%)	<0.01	322 (31.6%)	1455 (17.8%)	<0.01	848 (22.7%)	786 (20.9%)	0.16
Median OECD equivalised income/year	£14 689	£23 404	<0.01‡	£13 977	£20 048	<0.01‡	£17 459	£17 194	0.14‡
	(14 453 to £15 280)	(£23 114 to £23 620)		(£13 321 to £14 454)	(19 809 to £20 268)		(£17 258 to £17 761)	(£16 880 to £17 553)	
Mean number of children in household	3.1 (3.0 to 3.2)	2.3 (2.3 to 2.3)	<0.01†	2.7 (2.7 to 2.8)	2.5 (2.5 to 2.5)	<0.01†	2.6 (2.6 to 2.6)	2.6 (2.6 to 2.6)	0.76†
Mother ethnicity white British/Irish	298 (81.6%)	6695 (91.2%)	<0.01	815 (84.3%)	7145 (88.0%)	<0.01	3463 (86.3%)	3203 (83.1%)	<0.01
**Child health outcomes at T1 (7 years)**									
Overweight/obesity	104 (21.7%)	1524 (19.2%)	0.28	251 (22.9%)	1737 (18.8%)	<0.01	896 (21.1%)	896 (20.5%)	0.25
Problematic behaviour (teacher scored)	67 (26.9%)	693 (13.4%)	<0.01	145 (24.0%)	858 (15.2%)	<0.01	457 (18.6%)	308 (15.3%)	0.4
Problematic behaviour (parent scored)	116 (26.4%)	687 (9.8%)	<0.01	179 (19.4%)	997 (12.1%)	<0.01	590 (15.9%)	511 (13.9%)	0.04
Fair/poor general health score	29 (5.6%)	149 (2.0%)	<0.01	57 (5.3%)	210 (2.2%)	<0.01	158 (3.8%)	117 (2.6%)	0.02
Long-standing illness	108 (22.4%)	1381 (17.6%)	0.01	238 (21.8%)	1610 (17.9%)	<0.01	838 (20.5%)	750 (18.6%)	0.04
Bedwetting	69 (13.7%)	1145 (14.5%)	0.65	172 (15.2%)	1279 (14.4%)	0.24	633 (15.2%)	551 (13.7%)	0.02

Missing data: age of main respondent: n=445, maternal level of education: n=213, household employment: n=474, lone parent household: n=445, household income: n=484, number of children in household: n=445, country of residence: n=445, maternal ethnicity: n=699, weight status: n=543, Strengths and Difficulty Questionnaire (teacher scored): n=2182, Strengths and Difficulty Questionnaire (parent scored): n=603, general health score: n=456, long-standing illness: n=457, bedwetting: n=458.

NB: all percentages are survey weighted to account for study design and attrition.

*p Values calculated using χ^2^.

†p Values calculated using t-test.

‡p Values calculated using Mann-Whitney U test.

OECD, Organisation for Economic Co-operation and Development.

At T1, 31% (n=4056) of main respondents were below the poverty line (‘income poor’) and 44% (n=5671) reported difficulty managing financially. At T2, 21% (n=2700) were below the poverty line, 49% (n=6176) reported difficulty managing financially and 36% (n=4681) felt ‘worse off’.

**Figure 1:**
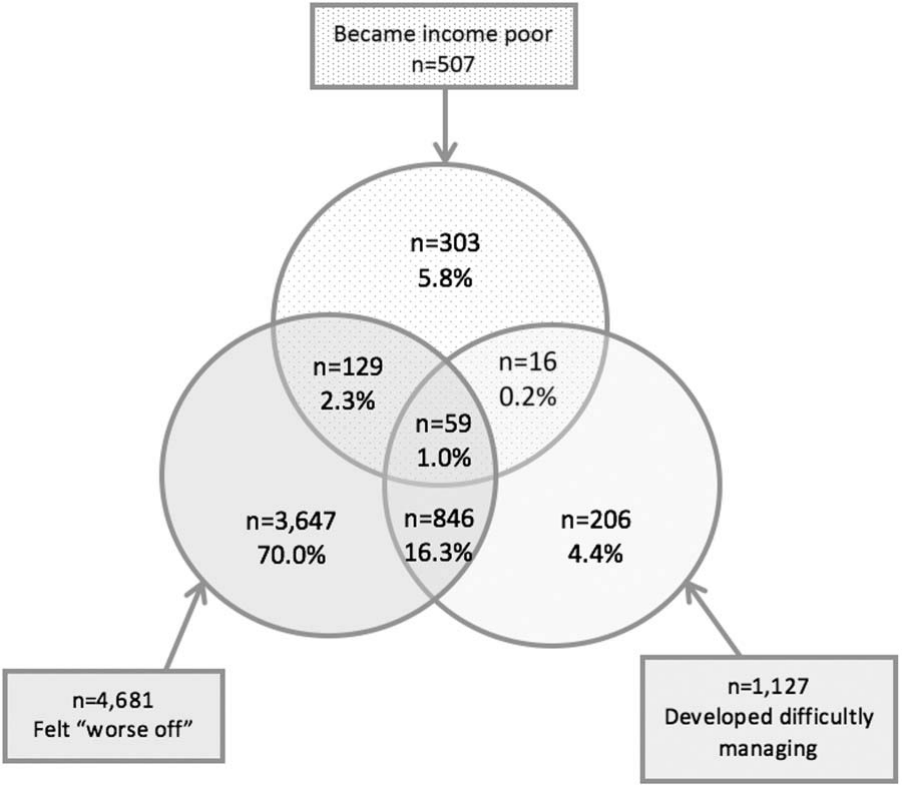
Venn diagram illustrating the overlap between the three measures of increased household financial strain, at T2 (n=5206).

Between T1 and T2, 39.2% (n=5206/13 005) of all households experienced an increase in FS. Those who ‘became income poor’ made up the smallest proportion (9.4%) and those who ‘felt worse off’, the largest (89.6%). Figure 1 summarises the overlap between the three measures of increased household FS at T2.

### Child health outcomes at 11 years (T2)

At T2, 29.3% (n=3530) of children were overweight or obese, 15.9% (n=1040) had problematic behaviour as scored by their teacher and 17.2% (n=1926) had problematic behaviour as scored by their parent, 3.6% (n=451) had a suboptimal general health score, 14.2% (n=1752) had a long-standing illness and 5.8% had bedwetting (n=698).

### Increased financial stain and new or continued poor child health at T2

#### Became income poor

Children in households which fell below the poverty line between T1 and T2 (‘became income poor’) were significantly more likely to be overweight or obese (RR 1.21, 1.04–1.42), have bedwetting (RR 1.54, 1.00–2.37) and problematic behaviour, regardless of whether it was scored by teacher (RR 2.05, 1.58–2.65) or parent (RR 2.20, 1.78–2.71), compared with those who remained above the poverty line (table 2). The increase risk of problematic behaviour remained statistically significant after adjustment for confounders, but was removed for overweight/obesity and bedwetting.

**Table 2:** Primary analysis: risk ratios (RR), adjusted risk ratios* (aRR) and CIs for poor child health outcomes at T2 (11 years), among those who experienced an increase in financial strain between T1 (7 years) and T2 (11 years), compared with reference groups

	Child health outcomes at T2																	
	Overweight/obesity			Problematic behaviour (teacher scored)			Problematic behaviour (parent scored)			Fair/poor general health score			Long-standing illness			Bedwetting		
	% (N)	RR (CI)	aRR* (CI)	% (N)	RR (CI)	aRR* (CI)	% (N)	RR (CI)	aRR* (CI)	% (N)	RR (CI)	aRR* (CI)	% (N)	RR (CI)	aRR* (CI)	% (N)	RR (CI)	aRR* (CI)
Became income poor	32.6 (149)	1.21	1.14	21.2 (52)	2.05	1.63	25.7 (112)	2.20	1.62	3.6 (24)	1.62	1.44	15.7 (73)	1.20	1.34	7.5 (35)	1.54	1.08
		(1.04 to 1.42)	(0.93 to 1.39)		(1.58 to 2.65)	(1.14 to 2.32)		(1.78 to 2.71)	(1.24 to 2.10)		(0.98 to 2.70)	(0.79 to 2.62)		(0.93 to 1.56)	(0.98 to 1.84)		(1.00 to 2.37)	(0.60 to 1.94)
Stayed non-poor	27.1 (1982)	–	–	10.8 (477)			11.7 (824)	–	–	2.2 (163)	–	–	13.0 (1011)	–	–	4.9 (385)	–	–
Developed difficulty managing	32.3 (332)	1.19	1.17	22.4 (122)	1.76	1.63	25.8 (236)	1.88	1.73	5.4 (61)	2.16	2.11	17.5 (189)	1.32	1.33	7.4 (74)	1.44	1.61
		(1.07 to 1.33)	(1.02 to 1.34)		(1.45 to 2.14)	(1.26 to 2.11)		(1.63 to 2.16)	(1.44 to 2.09)		(1.58 to 2.94)	(1.47 to 3.02)		(1.12 to 1.56)	(1.09 to 1.62)		(1.11 to 1.87)	(1.17 to 2.21)
Did not report difficulty managing	27.1 (2317)	–	–	12.6 (606)			13.7 (1126)	–	–	2.5 (224)	–	–	13.2 (1160)	–	–	5.1 (446)	–	–
Felt ‘worse off’	30.3 (1310)	0.99	1.04	17.8 (411)	1.14	1.15	19.4 (796)	1.13	1.27	4.7 (203)	1.44	1.74	15.7 (695)	1.22	1.33	6.1 (274)	1.30	1.27
		(0.92 to 1.06)	(0.96 to 1.14)		(0.97 to 1.33)	(0.95 to 1.40)		(1.01 to 1.27)	(1.09 to 1.49)		(1.13 to 1.84)	(1.26 to 2.39)		(1.09 to 1.38)	(1.14 to 1.55)		(1.03 to 1.63)	(0.94 to 1.71)
Felt ‘the same’	30.7 (1253)	–	–	15.9 (339)	–	–	17.2 (638)	2.2	–	3.3 (140)		–	12.9 (557)	–	–	4.7 (202)	–	–

NB: all percentages are survey weighted to account for study design and attrition.

*Risk ratios are adjusted for new lone parenthood (ie, two parent household at T1, 7 years and one parent household at T2, 11 years), ethnicity (main respondent white British/Irish, other), maternal level of education at 9 months (degree level or above) and parental age (continuous variable, years). Missing data: lone parenthood: n=1243, ethnicity: n=699, maternal education: n=213 and parental age: n=445.

#### Developed difficulty managing

Children in households which developed difficulty managing financially, between T1 and T2, were also significantly more likely to be overweight or obese (RR 1.19, 1.07–1.33), and have problematic child behaviour (teacher scored: RR 1.76, 1.45–2.14) (parent scored: RR 1.88, 1.63–2.16). In addition, they had a significantly increased risk of having a suboptimal general health score (RR 2.16, 1.58–2.94), a long-standing illness (RR 1.32, 1.12–1.56) and bedwetting (RR 1.44, 1.11–1.87) at T2, compared with those who remained financially comfortable (table 2). All RR remained statistically significant after adjustment for confounders.

#### Felt ‘worse off’

Children in households which felt ‘worse off’ at T2, compared with T1, were significantly more likely to have problematic behaviour (parent scored: RR 1.13, 1.01–1.27), a suboptimal general health score (RR 1.44, 1.13–1.84), long-standing illness (RR 1.22, 1.09–1.38) and bedwetting (RR 1.30, 1.03–1.63) at T2, compared with those who felt financially ‘the same’ (table 2). The increased risks of problematic behaviour, suboptimal general health score and long-standing illness remained statistically significant after adjustment for confounders.

### Subanalysis: households above the poverty line

In households which developed difficulty managing and were above the poverty line at T2, children were at a significantly increased risk of overweight and obesity (RR 1.28, 1.09–1.52), problematic behaviour (teacher scored: RR 1.71, 1.33–2.19) (parent scored: RR 1.96, 1.60–2.40), suboptimal general health score (RR 1.89, 1.19–3.00) and long-standing illness (RR 1.34, 1.07–1.66). All RRs remained significant after adjustment for confounders.

In households which felt worse off and were above the poverty line at T2, children were at a significantly increased risk of problematic behaviour (parent score: RR 1.30, 1.08–1.55), suboptimal general health score (RR 1.72, 1.20–2.45) and long-standing illness (RR 1.38, 1.19–1.61) (table 3). All RRs remained significant after adjustment for confounders.

**Table 3:** Subanalysis: risk ratios (RR), adjusted risk ratios* (aRR) and CIs for poor child health outcomes at T2, among those who experienced an increase in financial strain between T1 and T2, compared with reference groups and limited to households above the poverty line at T2

	Child health outcomes at T2 (limited to non-poor)																	
	Overweight/obesity			Problematic behaviour (teacher scored)			Problematic behaviour (parent scored)			Fair/poor general health score			Long-standing illness			Bedwetting		
	% (N)	RR (CI)	aRR* (CI)	% (N)	RR (CI)	aRR* (CI)	% (N)	RR (CI)	aRR* (CI)	% (N)	RR (CI)	aRR* (CI)	% (N)	RR (CI)	aRR* (CI)	% (N)	RR (CI)	aRR* (CI)
Developed difficulty managing	33.5 (230)	1.28	1.27	17.6 (76)	1.71	1.67	22.5 (146)	1.96	1.79	4.2 (34)	1.89	1.82	17.8 (138)	1.34	1.28	5.9 (45)	1.24	1.28
		(1.09 to 1.52)	(1.09 to 1.50)		(1.33 to 2.19)	(1.21 to 2.31)		(1.60 to 2.40)	(1.42 to 2.25)		(1.19 to 3.00)	(1.16 to 2.88)		(1.07 to 1.66)	(1.02 to 1.60)		(0.88 to 1.75)	(0.88 to 1.86)
Did not report difficulty managing	26.5 (1929)	–	–	10.8 (463)	–	–	11.7 (827)	–	–	2.2 (160)	–	–	12.9 (966)	–	–	4.8 (360)	–	–
Felt ‘worse off’	30.1 (1010)	1.05	1.06	15.0 (288)	1.18	1.22	16.6 (539)	1.30	1.34	4.2 (137)	1.72	1.74	16.1 (557)	1.38	1.37	5.6 (202)	1.29	1.27
		(0.95 to 1.16)	(0.96 to 1.18)		(0.99 to 1.42)	(0.97 to 1.54)		(1.08 to 1.55)	(1.12 to 1.62)		(1.20 to 2.45)	(1.21 to 2.50)		(1.19 to 1.61)	(1.16 to 1.62)		(0.95 to 1.75)	(0.92 to 1.74)
Felt ‘the same’	29.1 (915)	–	–	12.8 (228)	–	–	13.7 (397)	–	–	2.6 (83)	–	–	12.2 (416)	–	–	4.1 (142)	–	–

NB: all percentages are survey weighted to account for study design and attrition.

*Risk ratios are adjusted for new lone parenthood (ie, two parent household at T1, 7 years and one parent household at T2, 11 years), ethnicity (main respondent white British/Irish, other), maternal level of education at 9 months (degree level or above) and parental age (continuous variable, years). Missing data: lone parenthood: n=784, ethnicity: n=387, maternal education: n=121 and parental age: n=270.

## Discussion

In a nationally representative, contemporary cohort of children we found that increases in household FS, across the period of the Great Recession, were associated with new or continued poor child health and well-being. The findings are consistent with previous research which has shown an association between increased FS and poor health outcomes in adults^1^ and children.^2^ ^3^ However, this is the first study using longitudinal data to look at changes in household FS and a range of child health outcomes, over the period of the ‘Great Recession’. Additionally, we found that the negative health impacts of FS do not appear to be limited to those who are income poor.

Attrition is a common problem in cohort studies. More than one-quarter (28%) of the original cohort did not take part in the age 11 sweep. Households which did not take part in the surveys at age 7 and 11 years were more likely to be low-income, unemployed and single parents at earlier surveys, characteristics which were more common in households that experienced increase in FS. We used survey weights to account for attrition between sweeps, but it remains possible the weights did not fully account for any bias.

A further source of bias may be the self-reported nature of several of the measures used. For example, self-reported income can be unreliable or not accurately reflect how funds are distributed within the households.^15^ Furthermore, the definition of ‘income poverty’ is arbitrary and the equivalisation process used in the income measure does not account for inflation and housing costs; this may lead to an underestimation of the prevalence of FS. With the exception of overweight and teacher-reported problematic behaviour, all health measures were based on parental report. Although most used validated and/or widely employed questions, it is possible that respondents who experienced increases in FS would be more biased towards reporting poor child health than those who did not.

We looked at a variety of health outcomes and it is likely that the mechanisms through which increased FS might contribute to ill health vary. It is possible that pathways include a combination of material (eg, poor-quality housing, inability to afford ‘healthy foods’, difficulty accessing healthcare) and psychosocial factors (eg, strained domestic relationships and feelings of insecurity, inadequacy and stress). Future research could explore these potential mechanisms. Several studies have shown an association between socioeconomic disadvantage in childhood and the development of poor health, potentially years later.^17^ ^18^ Future research could explore the relationship between increased household FS and new unhealthy outcomes for children, in a larger data set and over longer periods of time.

Increased FS is associated with new or continued poor health among children, including among those who would not be considered ‘poor’ according to standard definitions. Therefore, measures of FS should not be limited to income. During times of widespread economic hardship, such as the ‘Great Recession’, measures to buffer families from FS may go some way to reducing the increased risks of poor health. Such measures might include ‘ring fencing’ specific welfare or public services, and should not be limited to those living in poverty.
